# Determinants of the cytosolic turnover of mitochondrial intermembrane space proteins

**DOI:** 10.1186/s12915-018-0536-1

**Published:** 2018-06-22

**Authors:** Lukasz Kowalski, Piotr Bragoszewski, Anton Khmelinskii, Edyta Glow, Michael Knop, Agnieszka Chacinska

**Affiliations:** 1 Centre of New Technologies, University of Warsaw, Warsaw, Poland; 2 International Institute of Molecular and Cell Biology, Warsaw, Poland; 3 Zentrum für Molekulare Biologie der Universität Heidelberg (ZMBH), Heidelberg, Germany; 4 Deutsches Krebsforschungszentrum (DKFZ), DKFZ-ZMBH Alliance, Heidelberg, Germany; 5 Institute of Molecular Biology (IMB), Mainz, Germany

**Keywords:** Ubiquitination, Protein import, Protein degradation, Proteasome, Mitochondria, Intermembrane space, Cox12

## Abstract

**Background:**

The proteome of mitochondria comprises mostly proteins that originate as precursors in the cytosol. Before import into the organelle, such proteins are exposed to cytosolic quality control mechanisms. Multiple lines of evidence indicate a significant contribution of the major cytosolic protein degradation machinery, the ubiquitin-proteasome system, to the quality control of mitochondrial proteins. Proteins that are directed to the mitochondrial intermembrane space (IMS) exemplify an entire class of mitochondrial proteins regulated by proteasomal degradation. However, little is known about how these proteins are selected for degradation.

**Results:**

The present study revealed the heterogeneous cytosolic stability of IMS proteins. Using a screening approach, we found that different cytosolic factors are responsible for the degradation of specific IMS proteins, with no single common factor involved in the degradation of all IMS proteins. We found that the Cox12 protein is rapidly degraded when localized to the cytosol, thus providing a sensitive experimental model. Using Cox12, we found that lysine residues but not conserved cysteine residues are among the degron features important for protein ubiquitination. We observed the redundancy of ubiquitination components, with significant roles of Ubc4 E2 ubiquitin-conjugating enzyme and Rsp5 E3 ubiquitin ligase. The amount of ubiquitinated Cox12 was inversely related to mitochondrial import efficiency. Importantly, we found that precursor protein ubiquitination blocks its import into mitochondria.

**Conclusions:**

The present study confirms the involvement of ubiquitin-proteasome system in the quality control of mitochondrial IMS proteins in the cytosol. Notably, ubiquitination of IMS proteins prohibits their import into mitochondria. Therefore, ubiquitination directly affects the availability of precursor proteins for organelle biogenesis. Importantly, despite their structural similarities, IMS proteins are not selected for degradation in a uniform way. Instead, specific IMS proteins rely on discrete components of the ubiquitination machinery to mediate their clearance by the proteasome.

**Electronic supplementary material:**

The online version of this article (10.1186/s12915-018-0536-1) contains supplementary material, which is available to authorized users.

## Background

Cellular protein synthesis by cytosolic ribosomes is a starting point of complex protein logistics. More than 50% of newly synthesized proteins need to be transported to their specific destinations from the site of their synthesis. Many specialized transport pathways cooperate to guarantee the precise distribution and maturation of proteins. Simultaneously, quality control pathways guard the process of protein transport and folding. Such quality control mechanisms react to the mislocalization or misfolding of proteins, with the goal of minimizing their deleterious effects on cellular function [1–3]. The requirement for sophisticated transport and quality control machinery is apparent for cellular organelles with well-defined boundaries. This is the case for mitochondria, which are essential organelles of eukaryotic cells that are the site for energy-converting oxidative phosphorylation, various anabolic and catabolic processes (e.g., amino acid and lipid metabolism), the production of iron-sulfur clusters, and are an integral part of cellular ion homeostasis and cellular signaling networks [4–8]. Mitochondria are defined by two membranes—mitochondrial outer membrane (OM) and mitochondrial inner membrane (IM)—that surround two distinct aqueous compartments: the intermembrane space (IMS) and the mitochondrial matrix [7, 9, 10]. To perform their functions, mitochondria need a specialized array of proteins. The best-characterized mitochondrial proteomes of the yeast *Saccharomyces cerevisiae* and humans contain 900–1500 different proteins [11–13]. The bulk of mitochondrial proteins is encoded by the nuclear genome, and these proteins are produced by cytosolic ribosomes. Such mitochondrial precursor proteins can constitute up to 15% of all proteins that are produced in the cytosol and require active transport into the organelle. To ensure accurate precursor protein targeting and proper maturation, several sorting and assembly pathways cooperate [14–20]. The first step of import, which is common to nearly all mitochondrial proteins, is crossing the mitochondrial OM by the translocase of the outer membrane (TOM) multi-subunit complex. After crossing the OM, proteins are routed further with their final destinations encoded in their amino acid sequence. Many proteins of the IMS follow the mitochondrial import and assembly (MIA) pathway, which combines protein import with oxidative folding. Mitochondria contain pathways to refold misfolded proteins or degrade damaged ones, thus providing quality control mechanisms for proteins that enter the organelle [21, 22]. Protein import into the mitochondria is in part co-translational [23–25]. However, the import of most mitochondrial precursor proteins can occur as a post-translational process. Such post-translationally imported precursors, from the time of their synthesis until they enter the mitochondrial compartment, are under the control of cytosolic quality control mechanisms [26]. These quality control mechanisms are crucial because the accumulation of unimported mitochondrial proteins severely impacts cellular protein homeostasis [27, 28]. When mitochondrial precursor proteins are mislocalized to the cytosol, they can initiate the reprogramming of cellular protein turnover. Cytosolic translation becomes constricted, and protein clearance is enhanced by an increase in assembly and activity of the proteasome [3, 28]. The proteasome is the major cytosolic machinery that degrades individual proteins. Several studies found that proteins that are destined to mitochondria are degraded by the proteasome both when import is defective and under physiological conditions [29–33]. Previously, we identified an entire group of mitochondrial proteins (i.e., clients of the MIA import pathway in the IMS) as substrates of the proteasome [31]. Proteasome-mediated degradation provides a surveillance mechanism for these IMS proteins and prevents their accumulation in an incorrect compartment. Notably, proteasomal degradation is not limited to situations in which protein import into mitochondria fails. A portion of IMS proteins is also continuously removed under physiological conditions when import machinery is fully functional. Thus, the proteasome adjusts the availability of IMS precursor proteins, directly affecting mitochondrial biogenesis [31]. Reaching the final location is one step in the maturation of mitochondrial proteins that must be accompanied by their proper folding. Damaged IMS proteins can be degraded within the organelle [30, 34–36]. However, unfolded proteins of the mitochondrial IMS can retro-translocate to the cytosol, thus becoming re-exposed to the proteasome [37]. Although the involvement of proteasomal degradation in shaping the IMS proteome is well evidenced, the exact mechanisms that govern this process remain obscure.

Proteins are marked for proteasomal degradation by the covalent attachment of ubiquitin [38–41]. Polyubiquitin chains are frequently built on the substrate, but even the attachment of a single ubiquitin can serve as a sufficient signal for the proteasomal degradation of small proteins [42]. Ubiquitination requires the concerted action of three types of enzymes. Ubiquitin is first activated by a ubiquitin-activating E1 enzyme and transferred to a ubiquitin-conjugating E2 enzyme. Subsequently, the E2 enzyme partners with an E3 ubiquitin ligase to conjugate ubiquitin to the recipient protein. E3 ubiquitin ligases form a large and diverse group. The diversity of E3 proteins is necessary because they serve as receptors for a broad range of substrates, thus providing specificity to the ubiquitination process. Ubiquitination signals can be removed by the antagonistic activity of deubiquitinating enzymes (DUBs), forming an additional level of control [43, 44]. We previously found that IMS proteins are ubiquitinated in vivo [31]. A better understanding of the mechanisms of IMS protein-specific degradation by the ubiquitin-proteasome system requires the identification of regulatory proteins that mediate this process. Specific features within a substrate protein provide internal signals, termed “degrons”, that determine recognition by E3 ubiquitin ligases and modulate degradation efficiency [45–47]. Many precursor proteins that are targeted to the IMS and are the substrates of the MIA pathway share a characteristic helix-loop-helix domain (CHCHD) fold. In a mature IMS-located protein, this fold is stabilized by disulfide bonds that are formed between cysteine residues. Cysteine residues that are involved in stabilizing the IMS protein structure are commonly arranged in evolutionarily conserved CX_3_C or CX_9_C motifs [48–50]. Remaining unknown, however, is whether common structural features of IMS proteins are part of degrons that determine their cytosolic turnover.

In summary, despite the clear involvement of the ubiquitin-proteasome system in the quality control of IMS proteins, little is known about the ways in which these proteins are selected for degradation. To better understand this process, we employed parallel approaches to monitor the cytosolic degradation of IMS proteins. Using a screening approach, we identified components of the ubiquitin-proteasome system that impact the stability of these proteins. We also unveiled that IMS proteins differ in their stability when exposed to the cytosolic environment. The Cox12 protein was the most rapidly degraded among the studied examples. We used Cox12 to investigate the way in which its internal features affect the ubiquitination process and turnover efficiency. Finally, we deciphered the antagonistic relationship of two processes: precursor protein import to mitochondria and precursor protein ubiquitination. Despite their structural similarities, we concluded that proteins of the IMS do not share a common ubiquitination mechanism. Redundant pathways appear to provide a ubiquitination signal that triggers proteasomal degradation. Importantly, ubiquitinated precursor proteins cannot be imported into mitochondria. However, under normal conditions, the import process outcompetes the ubiquitination reaction, allowing most of the precursor proteins to reach their destination within the organelle.

## Results

### Tandem fluorescent protein timer as a tool to investigate degradation of IMS proteins in vivo

**Fig. 1:**
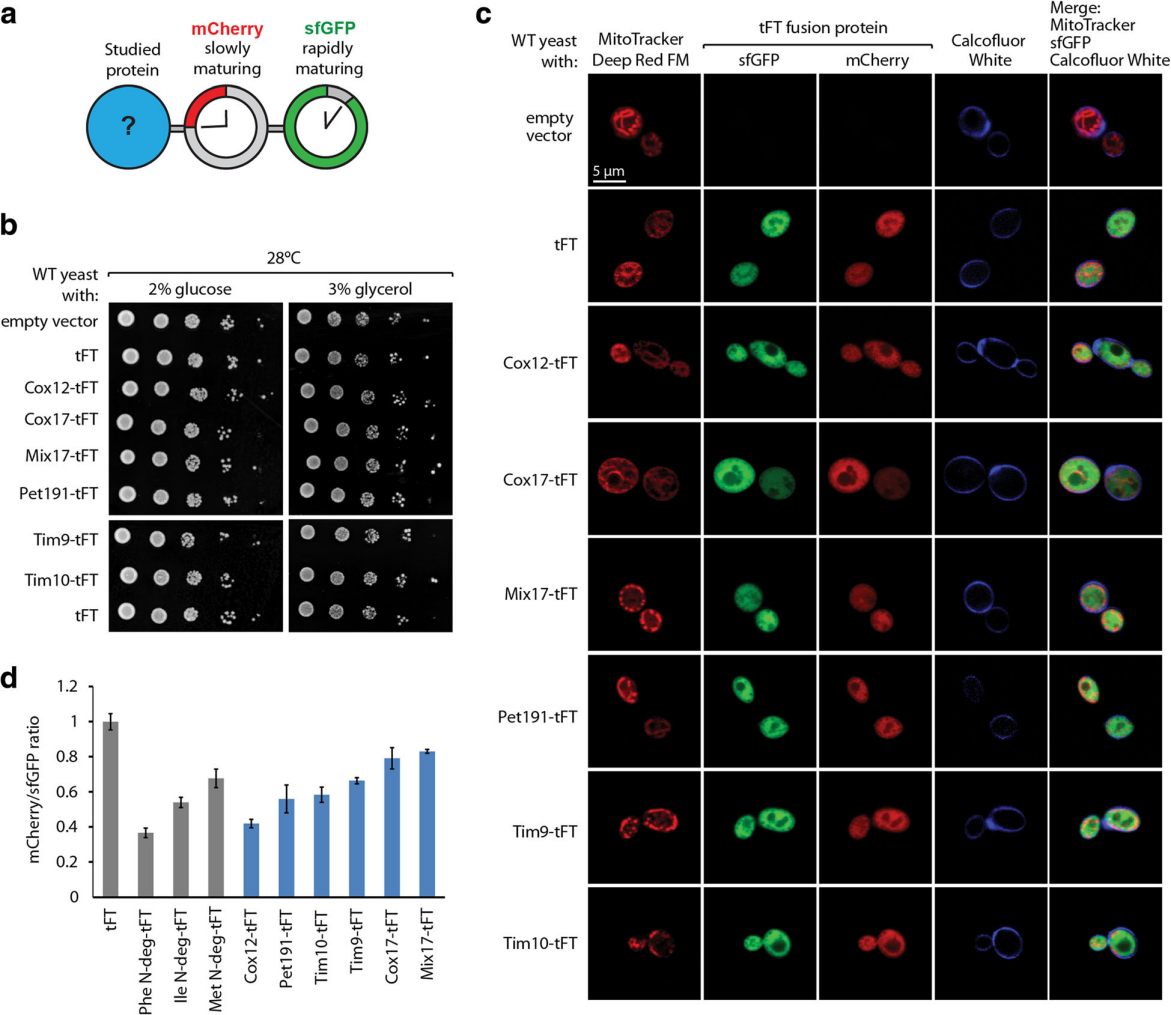
Diverse cytosolic stability of IMS proteins revealed by the tandem fluorescent protein timer approach. **a** Schematic illustration of a tandem fluorescent protein timer (tFT) fusion. The protein of interest is tagged with two fluorescent proteins with different maturation kinetics. The fluorescent signal ratio of slowly maturing mCherry protein and the rapidly maturing sfGFP protein reflects the half-life of the entire protein fusion. **b** Tenfold dilutions of WT cells that expressed the indicated plasmid-borne tFT fusions or an empty vector control were spotted on selective medium agar plates with either glucose or glycerol as the main carbon source. **c** Live confocal imaging of WT cells that expressed the indicated plasmid-borne tFT fusions and an empty vector control. Yeast were grown in minimal selective media with 3% glycerol at 24 °C. Prior to imaging, cells were stained with MitoTracker Deep Red FM and calcofluor white to label mitochondria and the cell wall, respectively. **d** Ratio of mCherry and sfGFP fluorescent signals measured in WT cells that expressed the indicated plasmid-borne tFT fusions. Cells were cultured in liquid selective media with 2% glucose at 28 °C. The ratio for the tFT alone was set to 1. The data are expressed as mean ± SEM. *n* = 6. ev, empty vector; N-deg, N-terminal degron; tFT, tandem fluorescent protein timer; WT, wild-type

To compare the cytosolic stability of proteins that are destined to the IMS, we employed tandem fluorescent protein timer (tFT) fusions as a tool to monitor protein turnover in vivo. In this approach, the protein of interest is expressed as a fusion with two fluorescent proteins: mCherry and superfolder green fluorescent protein (sfGFP; Fig. 1a) [51]. Rapidly folding and maturing sfGFP becomes fluorescent shortly after protein synthesis. In contrast, mCherry requires a longer time to develop fluorescence. The ratio of mCherry/sfGFP fluorescence intensities decreases as the degradation rate of mCherry-sfGFP fusions increases. A fraction of sfGFP from the tFT resists proteasomal degradation while mCherry is fully degraded [52]. This further decreases the mCherry/sfGFP fluorescence ratio with increasing degradation of the fusion protein [52]. The fluorescence of both proteins can be easily measured in vivo to allow direct comparisons. Any signals for degradation that are present in the protein of interest affect the tFT readout. Thus, the tFT that is fused to the C-terminus of a protein of interest allows the tracking of its degradation rate [51, 52]. We prepared tFT fusions of selected IMS proteins having either a CX_9_C motif (Cox12, Cox17, Mix17, and Pet191) or a CX_3_C motif (Tim9 and Tim10). The expression of such fusions did not affect the growth of a wild-type yeast strain, similar to the tFT alone (Fig. 1b). Next, we used confocal microscopy to monitor the subcellular localization of tFT-tagged IMS proteins (Fig. 1c). We used MitoTracker Deep Red FM dye as a marker for mitochondria and calcofluor white to stain the cell wall. Fluorescence signals of both sfGFP and mCherry were present throughout the cytoplasm, with nearly perfect co-localization of the two fluorophores as expected for fusion proteins. None of the tested tFT fusions was enriched within the cell areas that were stained with MitoTracker. Instead, all tFT fusions presented a cytoplasmic distribution pattern that was very similar to the tFT alone. This indicates that none of the tested IMS proteins were effectively targeted to mitochondria when they were tagged with the tFT. The lack of mitochondrial import of tFT-tagged MIA substrates can be likely attributed to the size and fold of the tFT and the limited import driving force [53, 54]. This assumption is supported by a genome-wide protein localization study in yeast, based on gene tagging with GFP, that did not assign MIA substrates to mitochondria, often indicating their cytosolic localization [55]. Importantly, the cytosolic localization of fusion proteins increased their exposure to the cytosolic quality control machinery. Next, we measured mCherry/sfGFP fluorescence ratios of tFT-tagged IMS proteins in vivo (Fig. 1d). We used the tFT itself (i.e., not fused with any protein) as a control because this fusion is highly stable within the cell [51]. We have also included three engineered tFT fusion proteins with N-terminal degrons that represent different rates of degradation dependent on the amino acid residue exposed at their N-terminus (phenylalanine, isoleucine, or methionine) [51, 56]. All tFT-tagged IMS proteins exhibited lower mCherry/sfGFP fluorescence ratios compared to the tFT alone. This indicates that all tested IMS proteins destabilized the tFT construct. Interestingly, the tested proteins differed broadly in their turnover, with Cox12 exhibiting the lowest mCherry/sfGFP ratio, indicating that this protein was the most unstable. Fluorescence ratios of mCherry/sfGFP measured for Cox12-tFT were similar to those measured for the most unstable control fusion with the phenylalanine N-terminal degron. Next in the order of increasing stability were Pet191 and Tim9 with measured fluorescence ratios comparable to the isoleucine N-terminal degron. Remaining IMS proteins exhibited fluorescence ratios in the range of the more stable N-degron tFT fusion with N-terminal methionine residue (Tim10, Cox17, and Mix17, Fig. 1d). Different rates of the cytosolic turnover of IMS proteins might indicate that signals for degradation that are present in these proteins differ with regard to either quality or quantity.

### Variant of Cox12 with mutated cysteine residues is a viable model to study degradation in the cytosol

**Fig. 2:**
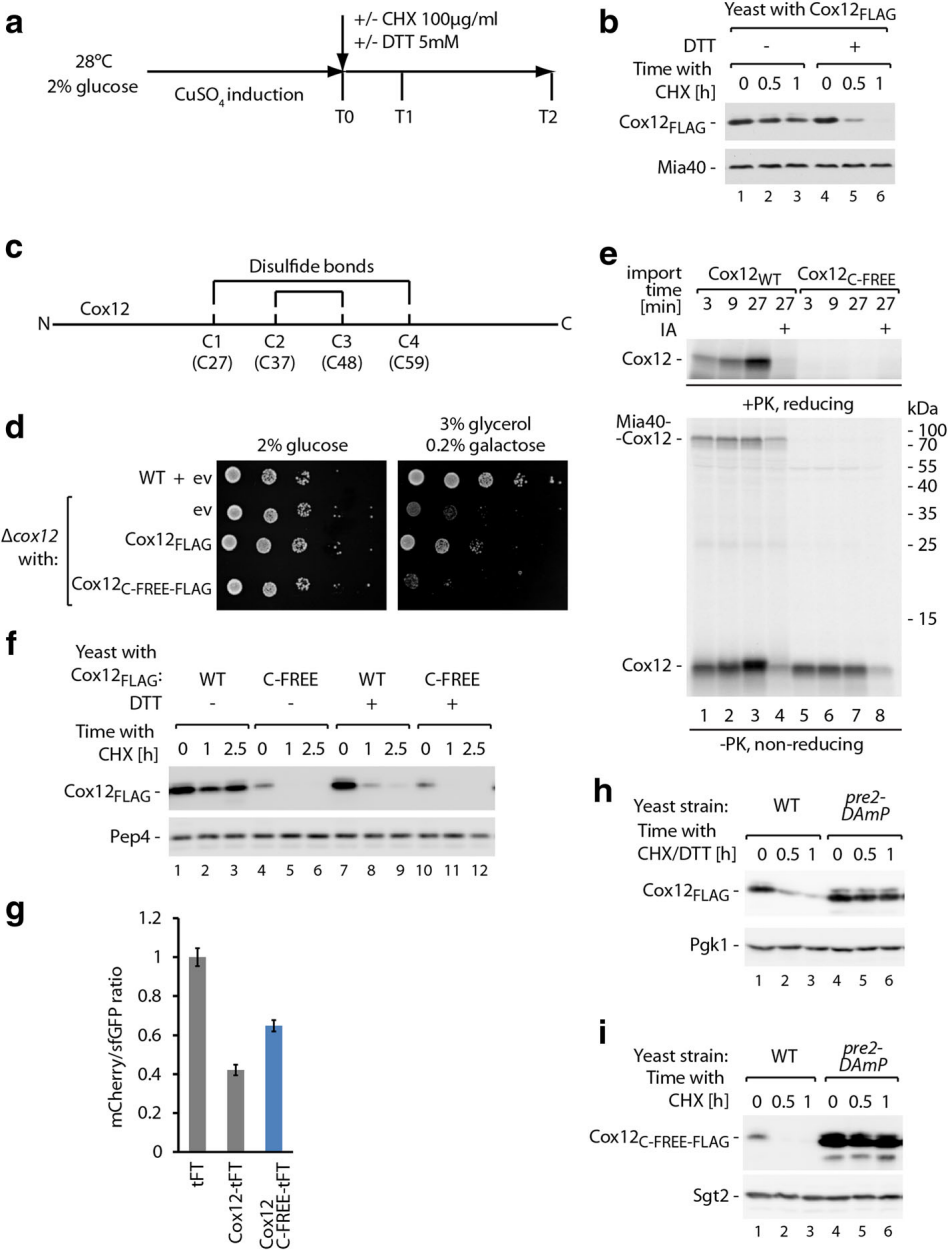
Disturbance of Cox12 protein oxidative folding triggers its rapid proteasome-mediated degradation. **a** Schematic illustration of the cycloheximide (CHX) chase experiments. **b** Degradation of Cox12_FLAG_ with or without 5 mM DTT treatment, tested using CHX chase. DTT was added in parallel to CHX as indicated. **c** Schematic representation of Cox12 protein with indicated positions of cysteine residues and disulfide bonds. **d** Growth test of WT and *Δcox12* yeast transformed with a plasmid that carried Cox12_FLAG_ or Cox12_C-FREE-FLAG_ under control of the galactose-inducible promoter or an empty vector control. Tenfold dilutions were spotted on selective minimal medium plates with a carbon source as indicated and grown at 28 °C. **e** Import of radiolabeled Cox12 or Cox12_C-FREE_ into mitochondria isolated from WT cells. The incubation times are indicated. Pretreatment with 50 mM IA was used as a negative control. **f** Degradation of Cox12_FLAG_ and Cox12_C-FREE-FLAG_, tested using CHX chase with or without DTT. **g** mCherry/sfGFP fluorescent signal ratio measured in WT cells that expressed plasmid-borne Cox12-tFT, Cox12_C-FREE_-tFT, and empty tFT. Cells were cultured in selective medium with glucose at 28 °C. The fluorescence ratio for the empty tFT was set to 1. Data are expressed as mean ± SEM, *n* = 6. Cox12-tFT and empty tFT data are the same as in Fig. 1d. **h**, **i** The degradation of Cox12_FLAG_ (**h**) or Cox12_C-FREE-FLAG_ (**i**) in WT or *pre2-DAmP* yeast, tested using CHX chase. In parallel to CHX, 5 mM DTT was added (**h**). **b**, **f**, **h**, **i** The plasmid-borne Cox12_FLAG_ was expressed using the copper-inducible promoter; yeast were cultured in selective medium with 2% glucose at 28 °C. Proteins were analyzed by SDS-PAGE and immunodetection (**b**, **f**, **h**, **i**) or autoradiography (**e**). CHX, cycloheximide; DTT, dithiothreitol; ev, empty vector; IA, iodoacetamide; PK, proteinase K; WT, wild-type

After determining that Cox12 was the most rapidly degraded IMS protein, we sought to examine its turnover without the relatively large tFT tag. We developed an assay based on the inducible overproduction of Cox12 tagged with a FLAG peptide (Cox12_FLAG_) followed by cycloheximide (CHX) treatment to inhibit cytosolic protein synthesis (Fig. 2a). Under such conditions, Cox12_FLAG_ was found to be relatively stable, with only a small decrease in protein levels after 1 h of CHX treatment (Fig. 2b, lanes 1–3). Thus, we sought to determine whether import and folding affected the cytosolic degradation of Cox12_FLAG_. The mitochondrial import and folding of MIA pathway substrates require disulfide bond formation [37, 48–50]. Thus, the reducing agent dithiothreitol (DTT) was added to the cell culture in parallel with CHX to inhibit oxidative folding. Our previous findings showed that treatment with a reducing agent led to the retro-translocation of reduced proteins from mitochondria. When CHX and DTT were applied in parallel, we observed much faster degradation of Cox12_FLAG_ (Fig. 2b, lanes 4–6). Treatment with DTT affects numerous aspects of cell functioning and might alter ubiquitination machinery, which is also thiol-based. Therefore, we developed an independent approach to monitor the cytosolic degradation of Cox12. Cox12 contains four cysteine residues that are arranged in two CX_9_C motifs that form two disulfide bonds in the mature protein (Fig. 2c). To prevent the oxidative import and folding of Cox12_FLAG_, we constructed the Cox12_C-FREE-FLAG_ mutant, in which all four cysteine residues were changed to serine residues. Cox12 is required for the assembly of cytochrome c oxidase. We tested whether the expression of Cox12_C-FREE-FLAG_ could rescue the inability of the *Δcox12* mutant to grow on respiratory media (Fig. 2d) [57]. Although non-mutated Cox12_FLAG_ could rescue the *Δcox12* growth defect, *Δcox12* yeast expressing Cox12_C-FREE-FLAG_ did not grow on the respiratory media, similar to the empty vector control. This suggests that Cox12_C-FREE-FLAG_ is not functional. Next, we investigated whether this lack of function is accompanied by a mitochondrial import defect as expected for this mutant. We employed an *in organello* import assay. Non-mutated Cox12 was effectively imported into isolated mitochondria in a time-dependent manner (Fig. 2e, upper panel, lanes 1–3). In the case of non-mutated protein, we also observed the formation of an import intermediate of Cox12 and Mia40 that was bound via a disulfide bond (Fig. 2e, see “non-reducing”), a characteristic of many MIA substrates. Further validating the import assay, Cox12 import was effectively blocked with iodoacetamide (IA) pretreatment, which irreversibly binds to cysteine residues and thus blocks the formation of disulfide bonds (Fig. 2e, lane 4). In contrast to wild-type Cox12, we observed complete inhibition of Cox12_C-FREE_ import and no intermediate formation with Mia40 (Fig. 2e, lanes 5–7). Therefore, we confirmed that Cox12_C-FREE_ cannot be imported into mitochondria and thus is likely to accumulate in the cytosol, resulting in its exposure to the ubiquitin-proteasome system. We subjected the mutant to a CHX chase experiment (Fig. 2f). The levels of Cox12_C-FREE-FLAG_ compared with Cox12_FLAG_ were lower from the beginning of the experiment, although the expression of both proteins was induced for the same length of time (Fig. 2f, compare lanes 1 and 4). Moreover, in contrast to Cox12_FLAG_, its cysteine residue-free variant was rapidly degraded (Fig. 2f). Degradation of Cox12_C-FREE-FLAG_ was not further affected by the addition of the reducing agent (Fig. 2f). To confirm that the rapid removal of the mutant protein is caused by its mislocalization to the cytosol and exclude the possibility of greater affinity of the degradation machinery that is caused by the mutations, we prepared a tFT fusion of Cox12_C-FREE_. When we compared mCherry/sfGFP fluorescence ratios of Cox12-tFT and Cox12_C-FREE_-tFT, we found a higher ratio for the C-FREE mutant, suggesting that the mutant degrades even slower than wild-type Cox12 when both proteins are located in the cytosol (Fig. 2g). Thus, the rapid degradation of Cox12_C-FREE_ can likely be attributed to its cytosolic mislocalization and not to greater affinity to the degradation machinery.

As reported previously, the degradation of Cox12 can be mediated by the proteasome [31, 37]. We sought to further validate that Cox12 is degraded by the proteasome in the cytosol. We compared the degradation rate in a wild-type yeast strain and in the *pre2-DAmP* strain that exhibits a decrease in proteasome activity [58]. In the *pre2-DAmP* strain, we observed the stabilization of both Cox12_FLAG_ during reductant treatment and Cox12_C-FREE-FLAG_ (Fig. 2h, i). Thus, we established an assay to monitor proteasome-mediated degradation of Cox12 that depends on mislocalization of the protein to the cytosol.

### Lysine residues are required for Cox12 ubiquitination

**Fig. 3:**
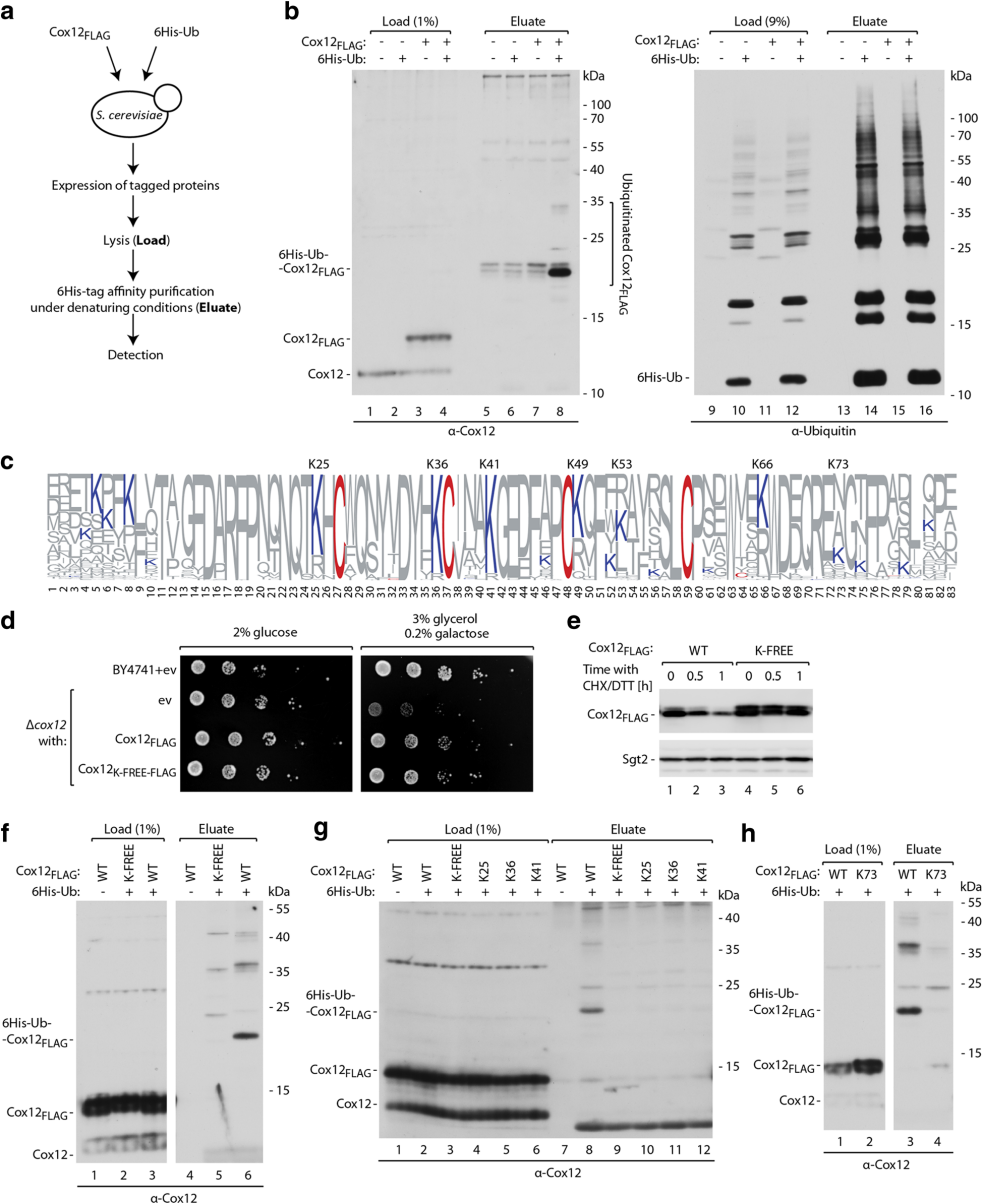
Lysine residues are required for Cox12 ubiquitination and proteasomal degradation. **a** Experimental scheme of 6His-tagged ubiquitin affinity purification. **b** Affinity purification of ubiquitinated Cox12_FLAG_ via 6His-ubiquitin. **c** Amino acid residue frequencies in Cox12 proteins among the selected fungal species. Amino acid numbering is according to *S. cerevisiae* protein. The data are presented in WebLogo format; cysteine and lysine residues are marked with red and blue, respectively. Positions of lysine residues that are present in *S. cerevisiae* Cox12 are indicated. **d** Growth test of WT and *Δcox12* yeast transformed with a plasmid that carried Cox12_FLAG_ or its mutant with all seven lysine residues mutated into arginine residues (Cox12_K-FREE-FLAG_) under the control of the galactose-inducible promoter and an empty vector control. Tenfold dilutions were spotted on selective minimal medium with a carbon source as indicated and grown at 28 °C. **e** Degradation of Cox12_FLAG_ and Cox12_K-FREE-FLAG_, tested using CHX chase experiments. The plasmid-borne Cox12_FLAG_ or Cox12_K-FREE-FLAG_ were expressed in WT cells using the copper-inducible promoter. Yeast were cultured in selective medium with 2% glucose at 28 °C. Samples were collected at the indicated time points, starting from the addition of CHX and DTT. **f** Affinity purification of ubiquitinated Cox12_FLAG_ and Cox12_K-FREE-FLAG_ via 6His-tagged ubiquitin. **g** Affinity purification of ubiquitinated Cox12_FLAG_, Cox12_K-FREE-FLAG_, and Cox12_K-FREE-FLAG_ variants with single lysine residues reintroduced at positions 25, 36, or 41. **h** Affinity purification of ubiquitinated Cox12_FLAG_ or its variant that possessed only a single lysine residue at position 73. **b**, **f**, **g**, **h** The plasmid-borne Cox12_FLAG_ variants were expressed in WT cells under control of the galactose-inducible promoter. Yeast were cultured in modified selective medium with 3% glycerol at 28 °C. **b**, **e**, **f**, **g**, **h** Proteins were analyzed by SDS-PAGE and immunodetection. 6His-Ub, 6His-tagged ubiquitin; CHX, cycloheximide; DTT, dithiothreitol; ev, empty vector; WT, wild-type

Protein tagging by ubiquitin allows for specific routing for degradation by the proteasome. Our previous results showed that Cox12, among other IMS proteins, can be ubiquitinated in vivo [31]. For a detailed investigation of Cox12 ubiquitination, we performed an assay for the affinity purification of ubiquitinated proteins (Fig. 3a). This assay confirmed that Cox12 is ubiquitinated in vivo (Fig. 3b, lane 8). We observed a strong band, the migration of which corresponded to the mass of Cox12_FLAG_ that was modified by one ubiquitin molecule, and additional weak bands that likely represented the attachment of more ubiquitin molecules. We detected ubiquitinated species only when both 6His-tagged ubiquitin and Cox12_FLAG_ were expressed simultaneously, thus confirming the specificity of the assay. Ubiquitin is typically attached to a lysine residue in the target protein [39, 41]. Thus, we sought to determine whether lysine residues are required for Cox12 ubiquitination. Cox12 contains seven lysine residues (Fig. 3c). We prepared a Cox12_K-FREE-FLAG_ mutant, in which all lysine residues were changed to arginine residues. Importantly, Cox12_K-FREE-FLAG_ complemented the lack of native Cox12 and enabled respiratory growth of the *Δcox12* strain (Fig. 3d). This indicates that the mutant protein can reach the IMS to perform its function. Next, we subjected Cox12_K-FREE-FLAG_ to the CHX chase experiment in presence of DTT (Fig. 3e). In contrast to non-mutated Cox12_FLAG_, the levels of Cox12_K-FREE-FLAG_ remained stable after the inhibition of protein synthesis (Fig. 3e, compare lanes 1–3 and 4–6). This supports the notion that lysine residues are the site for Cox12 ubiquitination and are required for its efficient removal by the proteasome. We used affinity purification of ubiquitin-conjugated species to test whether the Cox12_K-FREE-FLAG_ mutation prevents ubiquitination (Fig. 3f). We did not observe any bands that might represent ubiquitinated Cox12_K-FREE-FLAG_. Simultaneously, we could detect ubiquitinated wild-type Cox12 _FLAG_ (Fig. 3f, compare lanes 5 and 6). To determine the specific site of ubiquitin attachment, we investigated the conservation status of lysine residues among Cox12 from fungal species that are related to *S. cerevisiae* (Fig. 3c). Three lysine residues (K25, K36, and K41) are very conserved. Therefore, we tested whether any of these lysine residues might be sufficient to allow Cox12 ubiquitination. Based on Cox12_K-FREE-FLAG_, we created three mutants in which lysine residues were re-introduced at positions 25, 36, and 41, respectively. These Cox12 variants were tested for in vivo ubiquitination (Fig. 3g). The three mutants were efficiently expressed. However, similar to Cox12_K-FREE-FLAG_, we did not observe any ubiquitin conjugates. Thus, we established that the restoration of a single lysine residue (K25, K36, or K41) is insufficient to restore Cox12 protein ubiquitination. We also subjected the Cox12 amino acid sequence to in silico analysis with three different algorithms that were designed to predict ubiquitination sites (UbPred, CKSAAP_UBSITE, and UbiProber) [59–61]. All algorithms indicated a generally low probability of Cox12 ubiquitination, likely because of the small size of the protein. Despite this, all three algorithms indicated a poorly conserved lysine residue at position 73 as the most likely to be ubiquitinated. Thus, based on Cox12_K-FREE-FLAG_, we prepared a mutant with a re-introduced lysine residue at position 73. Also, in this case, we did not observe accumulation of the ubiquitinated form of the protein (Fig. 3h). One possibility is that other lysine residues that were not tested herein (i.e., at position K49, K53, or K66) are required for Cox12 protein ubiquitination. Another possibility is that lysine residues not only serve as the ubiquitination site but also are part of the recognition site. Therefore, more than one lysine residue may be required for Cox12 ubiquitination.

### Various components of the ubiquitin-proteasome system are engaged in the degradation of IMS proteins

**Fig. 4:**
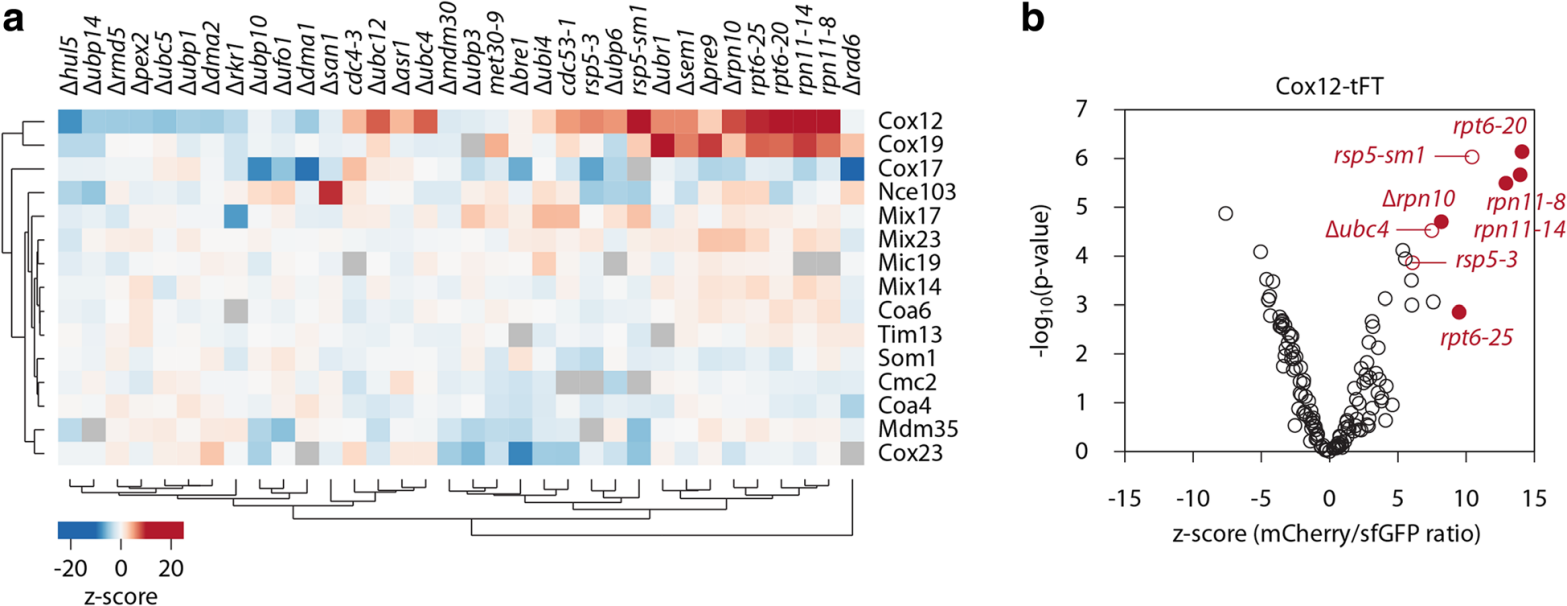
Screens for machinery involved in the degradation of IMS proteins. **a** Summary heat map of the screens for components of the ubiquitin-proteasome system that are involved in the degradation of the indicated tFT-tagged IMS proteins. Changes in protein stability (*z*-scores) are color-coded from blue (decrease) to red (increase). Only mutants that significantly affected the stability of at least one tFT-tagged protein (5% false discovery rate and |*z*-score| > 4) are shown. **b** Volcano plot of the screen results with Cox12-tFT, with *z*-scores for changes in protein stability on the *x*-axis and the negative logarithm of *p* values adjusted for multiple testing on the *y*-axis. 19S proteasome mutants are represented by filled circles

To gain further insights into the degradation of IMS proteins, we used a screening approach [62] to identify the factors that are involved in the degradation of tFT-tagged IMS proteins. Strains expressing IMS proteins that were chromosomally tagged with the tFT were crossed with an array of strains that carried mutations of individual components of the ubiquitin-proteasome system, including proteasome subunits, E2, E3, and DUB enzymes (Additional file 1: Table S1). Of the 18 chromosomally tFT-tagged substrates of the MIA pathway that were present in the tFT library, three (Cmc1, Mrp10, and Tim9) were removed from the screen because of growth/fluorescence defects when crossed with ubiquitin-proteasome mutants [62]. For the remaining 15 IMS proteins, the impact of each mutation on the stability of tFT fusions was examined with fluorescence measurements of colonies that were grown on the solid medium. We observed a diverse pattern of tFT responses that were specific to individual IMS proteins (Fig. 4a, Additional file 1: Table S1). Diverse factors appear to be responsible for the degradation of different IMS proteins, and no single factor appears to be involved in the degradation of all IMS proteins. Among the tested IMS proteins, Cox19 and Cox12 were consistently stabilized in mutants defective in proteasomal function. For several other tFT-tagged IMS proteins (Coa6, Mic19, Mix14, Mix17, Mix23, and Tim13), the mCherry/sfGFP fluorescence ratio also increased when the proteasomal function was perturbed but did not reach the threshold for significance (Fig. 4a). Proteasomal mutants (mutants of Rpn10, Rpn11, and Rpt6) were among those with the strongest stabilization of Cox12-tFT (Fig. 4b). Importantly, Cox12-tFT was also stabilized in the absence of the E2 ubiquitin-conjugating enzyme Ubc4 and in the mutants of the E3 ubiquitin ligase Rsp5 (Fig. 4b), suggesting that these two enzymes are potential regulators of cytosolic Cox12 turnover.

**Fig. 5:**
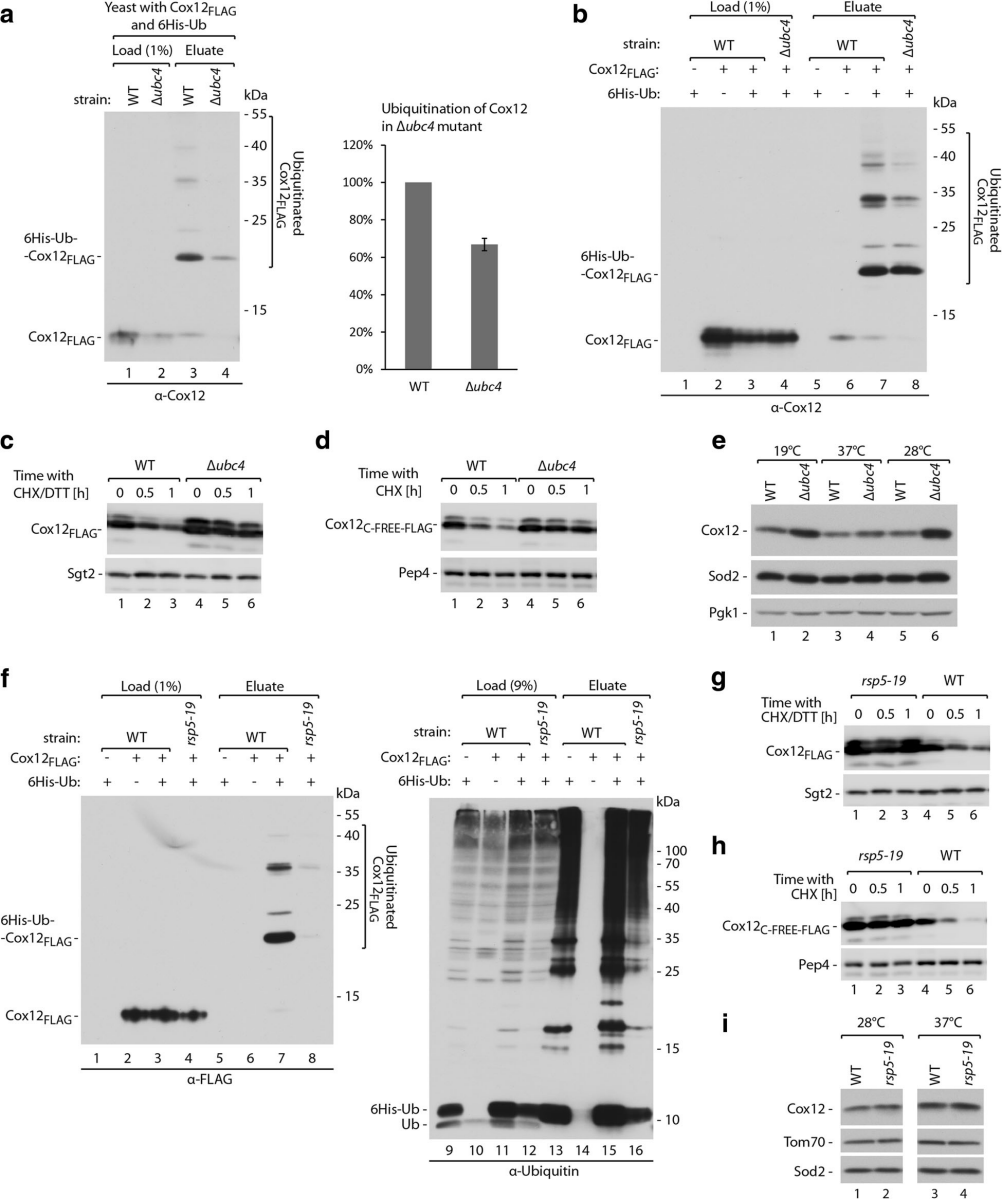
Involvement of the E2 ubiquitin-conjugating enzyme Ubc4 and the E3 ubiquitin ligase Rsp5 in Cox12 ubiquitination and degradation. **a**, **b** Affinity purification of ubiquitinated Cox12_FLAG_ via 6His-Ub from WT or *Δubc4* yeast cells. The plasmid-borne Cox12_FLAG_ was expressed under the control of a galactose-inducible promoter (**a**) or a copper-inducible promoter (**b**). Yeast were cultured at 28 °C in modified selective medium with 3% glycerol (**a**) or 2% glucose (**b**). Monoubiquitinated Cox12_FLAG_ levels were quantified and normalized to ubiquitin-free Cox12_FLAG_ levels from the load fractions (**a**). The level of normalized monoubiquitinated Cox12_FLAG_ from WT yeast was set to 100%. Data are expressed as mean ± SEM. *n* = 3. **c**, **d** Degradation of Cox12_FLAG_ (**c**) or Cox12_C-FREE-FLAG_ (**d**) in WT and *Δubc4* yeast, tested using CHX chase. The plasmid-borne Cox12_FLAG_ and Cox12_C-FREE-FLAG_ were expressed using the copper-inducible promoter. Yeast were cultured in selective medium with 2% glucose at 28 °C. **e** Cellular protein levels in WT and *Δubc4* yeast grown in minimal medium with 3% glycerol at indicated temperatures. **f** Affinity purification of ubiquitinated Cox12_FLAG_ via 6His-Ub in WT and *rsp5-19* yeast. The plasmid-borne Cox12_FLAG_ was expressed under the control of the copper-inducible promoter. Yeast were cultured in modified selective medium with 2% glucose at 37 °C. Degradation of Cox12_FLAG_ (**g**) or Cox12_C-FREE-FLAG_ (**h**) in WT and *rsp5-19* yeast. The plasmid-borne Cox12_FLAG_ or Cox12_C-FREE-FLAG_ were expressed under the control of the copper-inducible promoter. Yeast were cultured in selective medium with 2% glucose at 28 °C. **c**, **d**, **g**, **h** Samples were collected at the indicated time points, starting from the addition of CHX. In **c**, **g**, 5 mM DTT was added in parallel to CHX. **i** Cellular protein levels in WT and *rsp5-19* yeast grown in minimal medium with 3% glycerol at indicated temperatures. Proteins were analyzed by SDS-PAGE and immunodetection (all panels). 6His-Ub, 6His-tagged ubiquitin; CHX, cycloheximide; DTT, dithiothreitol; WT, wild-type

To validate the screening results, we tested whether components of the ubiquitination machinery (i.e., the E2 ubiquitin-conjugating enzyme Ubc4 and the E3 ubiquitin ligase Rsp5) that were identified in the screen were involved in Cox12 ubiquitination. We first compared the levels of ubiquitinated Cox12_FLAG_ in wild-type and *Δubc4* strains. When Cox12_FLAG_ production was driven by the *GAL10* promoter, we observed lower levels of both unmodified Cox12 and monoubiquitinated Cox12 in the *Δubc4* strain compared to the wild-type (Fig. 5a). Therefore, we used unmodified protein levels to normalize the observed monoubiquitinated Cox12_FLAG_ species. The quantification of monoubiquitinated Cox12_FLAG_ in the eluate fractions that were normalized to unmodified Cox12_FLAG_ levels showed a ~ 30% reduction of the monoubiquitinated form in the *Δubc4* strain (Fig. 5a, right panel). We performed a similar experiment using the *CUP1* promoter to drive Cox12_FLAG_ production, which results in higher protein levels. In this case, no difference in the levels of unmodified Cox12_FLAG_ was observed in the load fraction (Fig. 5b, lanes 3 and 4). In the eluate fractions, we observed a clear reduction of bands that represented poly- or multi-ubiquitinated Cox12_FLAG_ (Fig. 5b, lanes 7 and 8). These results indicate the involvement of the E2 ubiquitin-conjugating enzyme Ubc4 in Cox12 ubiquitination, but its role is not essential for the process. We also tested whether Ubc4 impacts the degradation of Cox12 using CHX chase experiments (Fig. 5c, d). In the *Δubc4* yeast strain, we observed the clear stabilization of both Cox12_C-FREE-FLAG_ and DTT-treated Cox12_FLAG_. These observations confirmed the important role of Ubc4 in Cox12 degradation by the proteasome. We also determined how the deletion of *UBC4* affects native Cox12 (Fig. 5e). The levels of native Cox12 increased in total protein extracts from *Δubc4* yeast compared with the wild-type strain. At the same time, the control proteins Sod2 and Pgk1 remained unaffected. Accumulation of Cox12 was visible at all tested growth temperatures: 19, 28, and 37 °C. This experiment provided further evidence for the involvement of Ubc4 in cytosolic degradation of Cox12.

Next, we investigated whether the E3 ubiquitin ligase Rsp5 is also involved in Cox12 ubiquitination. We employed an *rsp5-19* temperature-sensitive mutant strain [63, 64]. We first performed the purification of ubiquitinated Cox12_FLAG_ via 6His-ubiquitin in wild-type and *rsp5-19* genetic backgrounds (Fig. 5f). We observed similar levels of unmodified Cox12_FLAG_ in the load fractions. In the eluate fractions, a significant decrease in the levels of both mono- and polyubiquitinated Cox12_FLAG_ species was apparent in the *rsp5-19* mutant (Fig. 5f, compare lanes 7 and 8). This observation suggested the substantial involvement of Rsp5 in the ubiquitination of Cox12. However, we also observed lower levels of 6His-ubiquitin in the *rsp5-19* mutant compared with the wild-type strain in both the load and eluate fractions (Fig. 5f, right panel). Lower levels of ubiquitin may have contributed to the observed reduction of ubiquitinated forms of Cox12. Next, we tested the involvement of Rsp5 in Cox12 degradation using CHX chase experiments (Fig. 5g, h). In the *rsp5-19* mutant strain, we observed the stabilization of both Cox12_C-FREE-FLAG_ and DTT-treated Cox12_FLAG_. These observations supported the role of Rsp5 in Cox12 degradation by the proteasome. We also evaluated the levels of native Cox12 in the *rsp5-19* mutant (Fig. 5i). As opposed to the previous results, we did not observe increased levels of native Cox12 in the *rsp5-19* mutant. Thus, Rsp5 deficiency increased the accumulation of ectopically expressed Cox12 while the native protein was not significantly affected.

### Import impairment leads to increased ubiquitination and degradation of precursor proteins

**Fig. 6:**
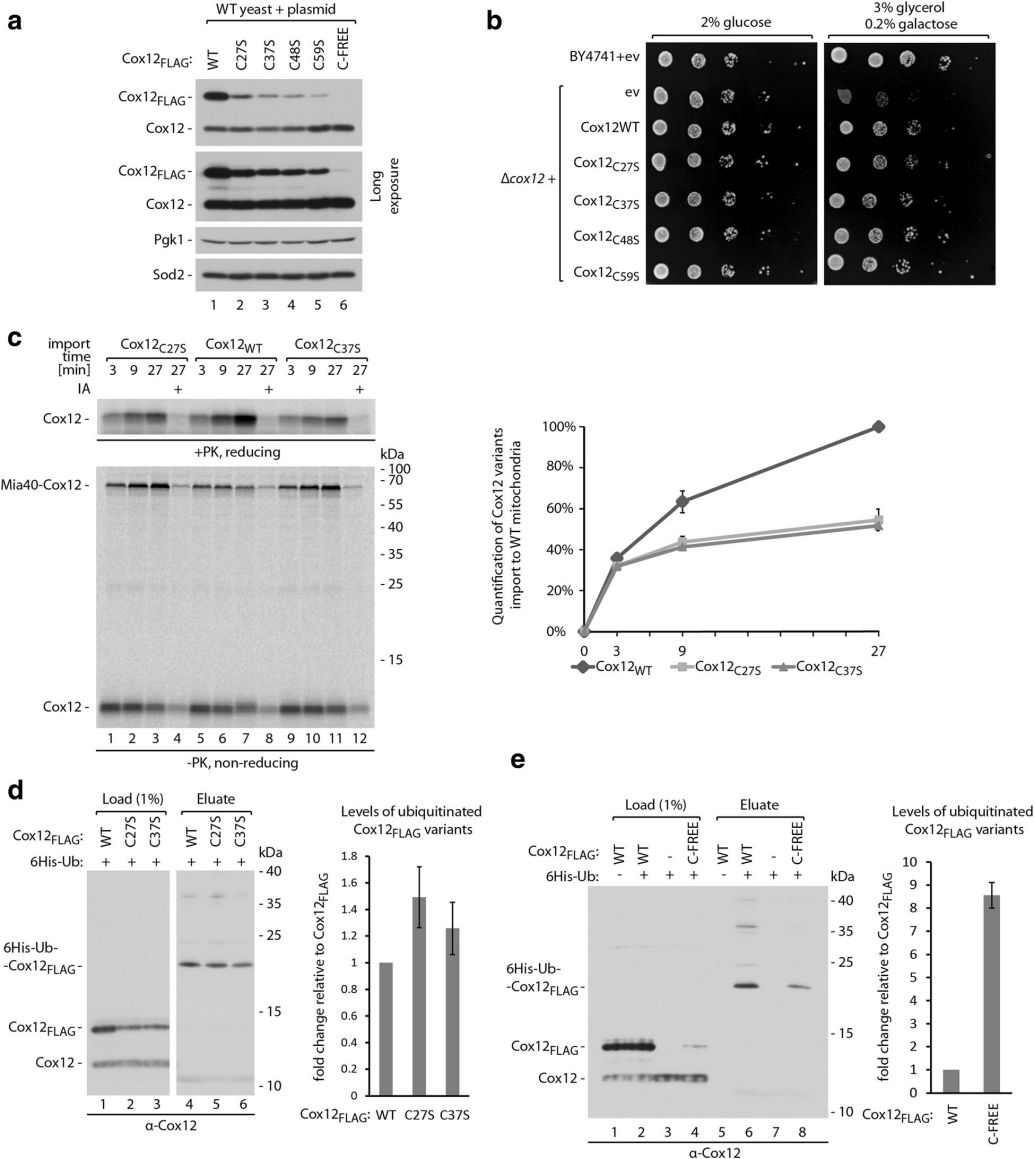
Less-efficient import to mitochondria results in increased protein ubiquitination and decreased cellular accumulation. **a** Cellular accumulation of Cox12_FLAG_, Cox12_C27S-FLAG_, Cox12_C37S-FLAG_, Cox12_C48S-FLAG_, Cox12_C59S-FLAG_, and Cox12_C-FREE-FLAG_ proteins in WT yeast. Yeast were cultured on selective minimal medium supplemented with glycerol as a carbon source at 28 °C. The expression of plasmid-borne Cox12_FLAG_ variants was driven by the galactose-inducible promoter. To induce expression, the medium was supplemented with 0.5% galactose for 6 h. **b** Growth test of WT and *Δcox12* yeast transformed with plasmids that carried Cox12_FLAG_ or one of its single cysteine residue mutants (C27S, C37S, C48S, C59S) and an empty vector control. Tenfold dilutions were spotted on selective minimal medium with a carbon source as indicated and grown at 28 °C. **c** Import of radiolabeled Cox12, Cox12_C27S-FLAG_, or Cox12_C37S-FLAG_ into mitochondria isolated from WT cells. Incubation times are indicated. Pretreatment with 50 mM IA was used as a negative control. The results were quantified, and the amount of WT Cox12 that was imported during 27 min of incubation was set to 100%. The data are expressed as mean ± SEM, *n* = 3. **d**, **e** Affinity purification of ubiquitinated Cox12_FLAG_ and Cox12_C27S-FLAG_, Cox12_C37S-FLAG_ (**d**), or Cox12_C-FREE-FLAG_ (**e**) from WT cells via 6His-Ub. Levels of monoubiquitinated Cox12_FLAG_ variants were quantified and normalized to ubiquitin-free Cox12_FLAG_ variants from the load fractions. The ubiquitinated Cox12_FLAG_ level was set to 100%. The data are expressed as mean ± SEM. *n* = 3. Yeast were cultured in liquid modified selective medium with 3% glycerol at 28 °C, and plasmid-borne Cox12_FLAG_ variants were expressed under the control of the galactose-inducible promoter. Proteins were analyzed by SDS-PAGE and immunodetection (**a**, **d**, **e**) or autoradiography (**c**). 6His-Ub, 6His-tagged ubiquitin; ev, empty vector; IA, iodoacetamide; PK, proteinase K; WT, wild-type

Mitochondrial proteins that are synthesized in the cytosol can be either imported into mitochondria or degraded by the ubiquitin-proteasome system. The kinetics of these two processes determine the ultimate abundance of mature proteins that accumulate in mitochondria. Under physiological conditions, protein import has an advantage over protein degradation. Therefore, the majority of precursor proteins can reach maturity inside the organelle. However, when protein import was blocked, we observed rapid degradation of Cox12 (Fig. 2b, f) [31]. To gain further insights into mutual relations of the protein import efficiency and protein degradation, we prepared four mutants of Cox12_FLAG_ in which single cysteine residues were exchanged for serine residues. We monitored protein levels after the induced expression of these mutants in wild-type cells using the *GAL10* promoter (Fig. 6a). The cellular levels of these four Cox12_FLAG_ single cysteine residue variants were lower compared with the non-mutated protein but higher than Cox12_C-FREE-FLAG_, pointing towards the intermediate stability of these mutants. Importantly, all four single cysteine residue mutants of Cox12_FLAG_ were functional as they complemented the loss of Cox12 and allowed for the respiratory growth of *Δcox12* yeast (Fig. 6b). This also indicates that there is no single specific cysteine residue in the Cox12 sequence that is essential for its import into mitochondria. However, removing any of the four cysteine residues prevents the formation of one of the two disulfide bonds that are present in mature Cox12 (Fig. 2c). We chose two mutants (C27S and C37S) to test how disruption of each of the two disulfide bonds affects import into mitochondria (Fig. 6c). Both Cox12_C27S_ and Cox12_C37S_ were imported ~ 50% less efficiently compared with the non-mutated protein (Fig. 6c, right panel). Additionally, higher levels of the import intermediates with Mia40 were observed for both mutants, indicating potential stalling during import (Fig. 6c, bottom panel). Next, we investigated the way in which less-efficient import affects the ubiquitination of these mutants. The quantification of affinity-purified monoubiquitinated forms of Cox12_FLAG_ that were normalized to the levels of Cox12_FLAG_ in the load fractions revealed that Cox12_C27S-FLAG_ and Cox12_C37S-FLAG_ mutants were 25–50% more ubiquitinated compared with non-mutated protein (Fig. 6d). Thus, slower protein import resulted in greater ubiquitination. Consequently, we expected that import-incompetent Cox12_C-FREE-FLAG_ would be even more ubiquitinated. Indeed, despite much lower levels of accumulated Cox12_C-FREE-FLAG_ compared with non-mutated Cox12_FLAG_, we purified similar amounts of ubiquitinated forms of Cox12 protein (Fig. 6e). After quantification and normalization, we estimated an almost tenfold increase in ubiquitination for this import-defective form of the protein (Fig. 6e, right panel).

### Protein ubiquitination blocks its mitochondrial import

**Fig. 7:**
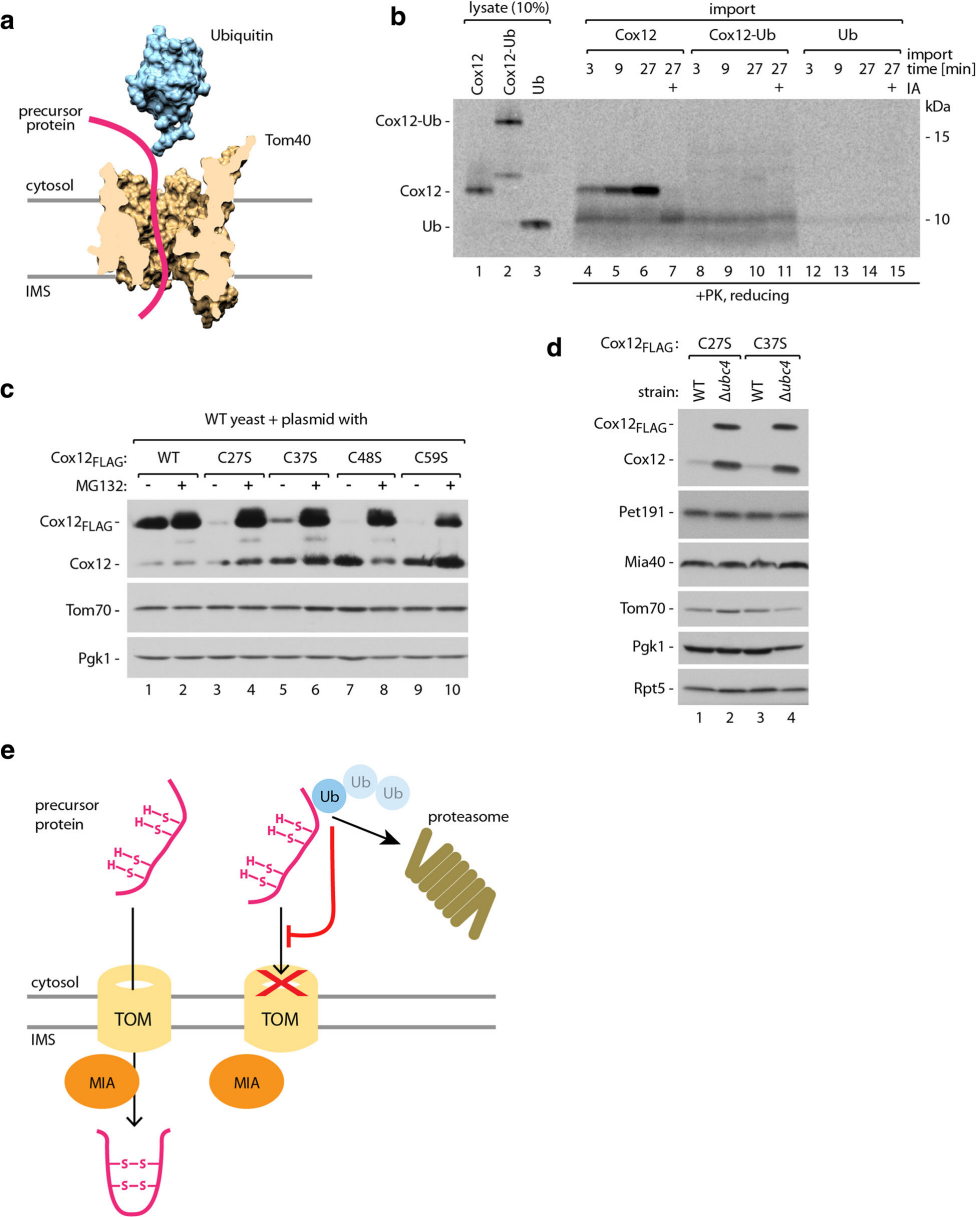
Cox12 protein ubiquitination prevents its import into mitochondria. **a** Juxtaposition of the three-dimensional density map of ubiquitin [PDB:1UBQ structure] and cross-section of the three-dimensional density map of the Tom40 protein-conducting channel based on homology modeling. **b** Import of radiolabeled Cox12, Cox12 head-to-tail fusion with ubiquitin (Cox12-Ub), and ubiquitin (Ub) into the mitochondria isolated from WT cells. The incubation times are indicated. Pretreatment with 50 mM IA was used as a negative control for MIA-dependent import. **c** Cellular accumulation of Cox12_FLAG_, Cox12_C27S-FLAG_, Cox12_C37S-FLAG_, Cox12_C48S-FLAG_, and Cox12_C59S-FLAG_ proteins in WT yeast with or without MG132 proteasome inhibitor treatment. Yeast were cultured in liquid modified selective medium with 3% glycerol at 28 °C; plasmid-borne Cox12_FLAG_ variants were expressed under the control of the galactose-inducible promoter. **d** Cellular accumulation of Cox12_C27S-FLAG_ or Cox12_C37S-FLAG_ proteins in WT and *Δubc4* yeast. Yeast were cultured in liquid selective medium with 3% glycerol at 28 °C; plasmid-borne Cox12_FLAG_ variants were expressed under the control of the galactose-inducible promoter. **e** Mitochondrial import of precursors of MIA substrate proteins is sensitive to ubiquitination. Ubiquitinated precursor proteins are rerouted from the mitochondrial import pathway to proteasomal degradation. Proteins were analyzed by SDS-PAGE and autoradiography (**b**) or immunodetection (**c**, **d**). IA, iodoacetamide; PK, proteinase K; Ub, ubiquitin; WT, wild-type

We sought to determine whether a ubiquitinated protein can escape degradation by the proteasome by getting imported into mitochondria. Proteins crossing the OM by the TOM translocase need to be largely unfolded [65–67]. A reasonable assumption is that folded ubiquitin may form spatial hindrance during protein transport through protein-conducting channel formed by Tom40 and would require at least partial unfolding. For illustration, we juxtaposed the three-dimensional cross-section of the Tom40 protein model and the ubiquitin structure (Fig. 7a) [66, 67]. The fully folded ubiquitin is substantially wider than the opening of the protein-conducting channel of Tom40. Considering the limited import-driving force of the MIA pathway, we hypothesized that ubiquitinated Cox12 protein cannot pass through the channel and thus cannot reach the mitochondrial IMS. To test this hypothesis, we created a construct that contained a head-to-tail fusion of Cox12 and ubiquitin. Such a fusion differs from the native condition, in which ubiquitin would be attached to one of the Cox12 lysine residues by the C-terminal glycine with an isopeptide bond. However, this model protein reflects the size restraints of natively monoubiquitinated Cox12 protein. In the *in organello* import experiment, Cox12-ubiquitin fusion did not enter the mitochondria, in contrast to the wild-type Cox12 (Fig. 7b, compare lanes 1–3 and 8–10). Also, free ubiquitin, which we tested in this experiment, did not present any affinity to isolated mitochondria (Fig. 7b, lanes 12–14). This result is consistent with our hypothesis that ubiquitination prevents the import of precursor proteins into mitochondria.

Altogether, our results showed that slower protein import into mitochondria leads to an increase in precursor protein ubiquitination, and ubiquitinated proteins cannot be imported into mitochondria. Both of these observations may explain the lower accumulation of Cox12 mutants with a partial import defect. We compared the accumulation of Cox12_FLAG_ variants under normal growth conditions and in the presence of the proteasome inhibitor MG132 (Fig. 7c). The accumulation of non-mutated Cox12_FLAG_ protein was only slightly increased by inhibition of the proteasome (Fig. 7c, lanes 1 and 2). At the same time, less effectively transported to mitochondria Cox12_FLAG_ mutants, in which one of the cysteine residues was changed to a serine residue, responded very strongly to proteasome inhibition (Fig. 7c, lanes 3–10). The cellular levels of all of these mutants were significantly lower compared with the non-mutated protein under normal growth conditions but strongly increased upon proteasome inhibition up to the level of non-mutated protein. To test whether similar accumulation rescue would be visible when protein ubiquitination is compromised, we expressed Cox12_C27S-FLAG_ and Cox12_C37S-FLAG_ in wild-type and *Δubc4* mutant cells. We observed an increase in the accumulation of mutant Cox12_FLAG_ proteins in *Δubc4* mutant cells, similar to proteasome inhibition (Fig. 7d). Together, these results suggest direct competition between protein import to mitochondria and protein ubiquitination (Fig. 7e).

## Discussion

Our previous studies identified proteins from the mitochondrial IMS as substrates of the cytosolic ubiquitin-proteasome system, but determinants of this process have remained unknown [31, 37]. The present study conducted an in-depth analysis of the cytosolic degradation of mitochondrial IMS proteins. We established a tandem fluorescent protein timer technique to monitor the cytosolic turnover of IMS proteins. Using this tool, we unveiled that proteins that are destined to the IMS differ in their stability while exposed to the cytosolic environment. This can be attributed to the varying affinity of IMS proteins to the ubiquitination machinery or proteasome as well as import efficiencies. Among the IMS proteins that were tested, Cox12 was the most rapidly degraded in the cytosol. Thus, we used Cox12 to decipher internal features that constitute the degron of this protein. We considered the possibility that ubiquitin is attached to cysteine residues of MIA substrate proteins because such a non-canonical manner of ubiquitination was reported for other proteins [68, 69]. This was plausible because of the presence of highly conserved cysteine residues in MIA substrate proteins. In the cytosol, these cysteine residues remain reduced and thus available for modification. However, our results exclude the contribution of ubiquitination on cysteine residues to cytosolic turnover of Cox12. A variant of Cox12 in which all of the cysteine residues were changed to serine residues was still efficiently ubiquitinated and processed by the proteasome. We also found that lysine residues were essential for Cox12 ubiquitination and degradation by the proteasome. The Cox12 variant in which all of the lysine residues were substituted by arginine residues was not ubiquitinated and was significantly more stable than the native protein variant. Thus, Cox12 ubiquitination occurred in a canonical way, but we did not find a single lysine residue that would be sufficient to sustain Cox12 ubiquitination. This could indicate that lysine residues not only serve as ubiquitination sites but also are a part of the sequence motif that is recognized by ubiquitination machinery. In such case, more than one lysine residue might be required for Cox12 ubiquitination. Another explanation could be that Cox12 is ubiquitinated on one of the lysine residues with low evolutionary conservation.

Using a systematic screening approach, we uncovered effects of various ubiquitin-proteasome system components on the cytosolic stability of specific IMS proteins. In particular, two proteins (Cox12 and Cox19) exhibited marked changes in tFT fluorescence, indicating their stabilization in proteasome deficient strains. For many other IMS proteins, we observed a similar increase in mCherry/sfGFP fluorescence ratios, albeit in a less well-defined manner. This may be partially attributable to the higher stability of these IMS proteins, because rapid turnover might be a factor that increases the sensitivity of tFT fusions. Moreover, the screens were performed at 30 °C, a semi-restrictive temperature for the temperature-sensitive mutants in proteasome subunits (e.g., *rpt6* and *rpn11* mutants). Therefore, some proteasome substrates are likely not strongly affected in these mutants in our screens. Surprisingly, despite the structural similarities of IMS proteins, no single universal factor is involved in the degradation of all IMS proteins. Conversely, the screening results showed that diverse ubiquitin-proteasome system components mediate the degradation of specific IMS proteins. Importantly, we verified the screening results for Cox12, confirming the role of two components of ubiquitination machinery: E2 ubiquitin-conjugating enzyme Ubc4 and E3 ubiquitin ligase Rsp5 [70, 71]. These two enzymes were previously shown to cooperate with each other, thus supporting the possibility of their joint action in Cox12 ubiquitination [72–74]. Ubc4 was previously shown to be involved in the cytosolic degradation of apo-iso-1-cytochrome *c*, another protein that is directed to the IMS [32]. Rsp5 was also previously reported to be involved in maintaining the mitochondrial proteome and to be recruited to mitochondria upon stress stimuli [75–78]. The results of our biochemical experiments confirmed the decrease in the ubiquitination of Cox12 in yeast strains without the *UBC4* gene or with Rsp5 activity impaired by genetic mutation (*rsp5-19*). However, in both cases, ubiquitination was abolished only partially. We also observed an increase in the stability of overproduced FLAG-tagged Cox12 in both mutant strains, but the levels of native Cox12 protein were visibly affected only in the *Δubc4* mutant. These observations suggest the redundancy of ubiquitination components. The activity of Ubc4 was reported to be partially redundant with its paralog Ubc5 [70, 73]. However, our screening results did not indicate the involvement of Ubc5 in Cox12 degradation (Fig. 4a, Additional file 1: Table S1). Thus, the partial reduction of Cox12 ubiquitination in the *Δubc4* strain can be explained by the existence of other redundant E2 ubiquitin-conjugating enzymes. Similarly, the fact that we observed an increase in the accumulation of native Cox12 protein in *Δubc4* but not in the *rsp5-19* mutant can indicate that E3 ubiquitin ligases are also redundant. Another possible explanation is that dependence on Rsp5 is connected with prolonged exposure of Cox12 to the cytosolic environment that results from expression of tagged protein from the plasmid. Notably, Cox12 does not contain a PY motif that is characteristic of client proteins that directly interact with Rsp5 ligase [79]. Thus, an additional adaptor protein that bridges Cox12 and E3 ubiquitin ligase is likely involved in the ubiquitin conjugation process. Not all such adaptor proteins were included in our screening approach and may require further investigation. For some IMS proteins, redundancy between different ubiquitination pathways could explain the observed lack of stabilization in any single UPS mutant.

Our screening results and the observed wide stability spectrum of IMS protein tFT fusions indicate that these proteins contain different and specific signals for degradation (i.e., degrons). Thus, we could reject the hypothesis that common features of MIA substrate proteins (i.e., conserved cysteine residues motifs within α-helix regions) may serve as shared, universal recognition traits for E3 ubiquitin ligase. Instead, our results indicate that IMS proteins rely on various recognition components that are responsible for their ubiquitination. Importantly, our protein turnover measurements rely on tFT fusions with independent evidence only for Cox12. Thus, further studies would be needed to verify if tagging with tFT influenced IMS proteins stability.

Another important issue is the way in which the ubiquitination of mitochondrial precursor proteins affects their import into the organelle. Mitochondrial proteins comprise a substantial part of the cellular ubiquitin-conjugated proteome [80]. Such ubiquitinated proteins can localize to mitochondria, and some were found localized inside the organelle [81, 82]. A recent report described a case of ubiquitination in the mitochondrial matrix, but free ubiquitin was not detected in isolated mitochondria [82]. Our results also demonstrate that free ubiquitin is not imported into isolated mitochondria or does not bind to the organelle surface. Importantly, proteins need to be largely unfolded to pass the TOM translocase, because most folded domains would not fit into the protein-conducting channel formed by Tom40 [65–67]. This is also the case for IMS proteins which once folded cannot traverse Tom40 translocase [37, 83]. Ubiquitin folds very stably, and a large pulling force is required for its mechanical unfolding [84]. Presumably, protein import into the matrix that is driven by ATP hydrolyzing PAM motor would provide sufficient energy to unfold ubiquitin conjugated to the precursor protein in transit. However, import via the MIA pathway is initially based only on the affinity of precursor proteins to the import machinery [36, 53, 54]. Given the limited driving force of IMS protein import, we assumed that even a single ubiquitin attached to the precursor would block its mitochondrial import, similar to the observed for tFT fusions. Our results confirmed this hypothesis with lack of mitochondrial import of Cox12-ubiquitin fusion. The import defect could not be attributed to simple size increase of the precursor protein as our previous results indicate that doubling the size of MIA substrate protein did not affect its import into mitochondria [37]. Thus, it is fair to assume that lack of import was a result of ubiquitin fold. Remaining unclear is whether the spatial hindrance that is formed by ubiquitin is a sole cause of import blockade or whether an additional mechanism prevents the import of ubiquitinated proteins, similar to the mechanism that was recently proposed for IM integral proteins [33].

We previously proposed that the proteasome regulates the composition of the IMS proteome. The present study broadens this observation to include also protein ubiquitination which was shown to directly affect the ability of the precursor protein to be imported into the organelle. Mitochondrial protein import via the MIA pathway is likely slower than import via the TIM23 translocase [85]. Still, under normal conditions, import of MIA substrates appears to have a kinetic advantage over the ubiquitination process, and only a minor portion of precursor proteins becomes ubiquitinated. However, with the decreasing efficiency of protein import, more precursor proteins may become ubiquitinated and thus rerouted from the import pathway to proteasomal degradation. Evidence of a tight connection between mitochondria and the ubiquitin-proteasome system is increasing, and our mechanistic understanding of the interplay between these two cellular systems continues to advance. Nonetheless, there are still unanswered questions. Most of our current analyses were based on Cox12 protein and its derivatives. Our results indicate that different IMS proteins can be tagged for degradation by separate ubiquitination components. Also, the internal features of IMS proteins that affect their degradation (i.e., degrons) are likely distinct. Thus, some of the observations that were made for Cox12 may be specific to this protein. However, effective import blockade that was caused by precursor protein modification with ubiquitin is likely universal to other MIA substrate proteins of the IMS and should be considered as a contributor to mitochondrial protein homeostasis.

## Conclusions

Altogether, our results substantiate the role of the ubiquitin-proteasome system in the regulation and quality control of mitochondrial IMS proteins. Notably, ubiquitination not only provides the signal targeting IMS proteins for degradation by the proteasome but also directly prohibits their mitochondrial import. Therefore, ubiquitination limits the availability of precursor proteins for organelle biogenesis. Remarkably, common sequence motifs that are characteristic for the substrates of the MIA import pathway do not constitute universal degron recognized by the cytosolic protein degradation machinery. Instead, individual proteins must contain specific signals for degradation as they rely on discrete components of the ubiquitination machinery to mediate their clearance.

## Methods

### Yeast strains, culture conditions, and plasmids

The *Saccharomyces cerevisiae* strains that were used in this study are listed in Table 1. Yeast were grown in full YPD or YPG media (1% yeast extract, 2% peptone, and 2% glucose or 3% glycerol, respectively) or in minimal media that contained 0.5% ammonium sulfate and 0.17% yeast nitrogen base, supplemented with suitable nutrients and a carbon source as indicated. For proteasome inhibition with MG132 (75 μM), the modified minimal medium without ammonium sulfate but with 0.1% proline and 0.003% sodium dodecyl sulfate (SDS) was used as described previously [31]. To induce the *GAL10* promoter in liquid culture, 0.5% galactose was added. To induce the *CUP1* promoter, 100 μM CuSO_4_ was added. To block protein synthesis, the cultures were supplemented with CHX to a final concentration of 100 μg/ml. Where indicated, 5 mM DTT was added in parallel with CHX to interfere with oxidative protein folding. Samples were collected after the indicated incubation times and flash frozen until preparation of total protein extracts that were subjected to further analysis. Plasmids that were used to express the proteins of interest are listed in Table 2. Yeast cells were transformed according to a standard procedure. For the growth test, selective minimal medium that contained 2.5% (*w*/*v*) agar was used. Where indicated, the addition of 0.2% galactose was used to induce the expression of proteins under control of the *GAL10* promoter.

**Table 1:** Yeast strains used in this study

Strain (lab ID no.)	Genotype	Reference
YPH499 (524)	*MAT*a, *ade2-101*, *his3-Δ200*, *leu2-Δ1*, *ura3-52*, *trp1-Δ63*, *lys2-801*	[101]
BY4741 (755)	*MAT*a, *his3Δ 1*, *leu2Δ 0*, *met15Δ 0*, *ura3Δ 0*	Euroscarf
*Δcox12* (722)	*MAT*a, *his3Δ 1*, *leu2Δ 0*, *met15Δ 0*, *ura3Δ 0*, *YLR038c::kanMX4*	Euroscarf
*Δubc4* (1007)	*MAT*a, *his3Δ 1*, *leu2Δ 0*, *met15Δ 0*, *ura3Δ 0*, *YBR082C::kanMX4*	Open Biosystems
*pre2-DAmP* (1051)	*MAT*a, *his3Δ 1*, *leu2Δ 0*, *met15Δ 0*, *ura3Δ 0*, *YPR103W::YPR103W-DAmP-kanMX4*	Dharmacon
MHY501 (794)	*MATα his3-Δ200 leu2-3112 ura3-52 lys2-801 trp1-1*	[102]
*rsp5-19* (796)	*MATα his3-Δ200 leu2-3112 ura3-52 lys2-801 trp1-1*, *rsp5::rsp5-19 (P418L)*	[63]

**Table 2:** Plasmids used in this study

Name (lab ID no.)	Backbone and description	Reference
pRS414	pRS414	[101]
pYEp96–6HIS-Ub (161)	pYEp96-CUP1^PR^-6His-tagged ubiquitin	[103]
pRS425	pRS425	[101]
pLK1 (589)	pRS425-CUP1^PR^-6His-Ub	This study
pESC-URA	pESC-URA	Agilent
pAG3 (55)	pESC-URA-GAL10^PR^-Cox12_FLAG_	[31]
pPB36.1 (471)	pESC-URA-CUP1^PR^-Cox12_FLAG_	This study
pEG1 (103)	pESC-URA-GAL10^PR^-Cox12_C27S-FLAG_	This study
pEG2 (104)	pESC-URA-GAL10^PR^-Cox12_C37S-FLAG_	This study
pEG3 (105)	pESC-URA-GAL10^PR^-Cox12_C48S-FLAG_	This study
pEG4 (106)	pESC-URA-GAL10^PR^-Cox12_C59S-FLAG_	This study
pLK2 (590)	pESC-URA-GAL10^PR^-Cox12_C-FREE-FLAG_	This study
pPB37.1 (472)	pESC-URA-CUP1^PR^-Cox12_C-FREE-FLAG_	This study
pPB32 (467)	pESC-URA-GAL10^PR^-Cox12_K-FREE-FLAG_	This study
pPB40.1 (613)	pESC-URA-CUP1^PR^-Cox12_K-FREE-FLAG_	This study
pLK3 (591)	pESC-URA-GAL10^PR^-Cox12_K25-FLAG_	This study
pLK4 (592)	pESC-URA-GAL10^PR^-Cox12_K36-FLAG_	This study
pLK5 (593)	pESC-URA-GAL10^PR^-Cox12_K41-FLAG_	This study
pPB33 (468)	pESC-URA-GAL10^PR^-Cox12_K73-FLAG_	This study
pRS415	pRS415	[101]
pPB19.1 (200)	p415-GDP^PR^-mCherry-sfGFP	This study
pPB20.1 (201)	p415-GDP^PR^-Cox12-mCherry-sfGFP	This study
pPB24.1 (385)	p415-GDP^PR^-Cox12_C-FREE_-mCherry-sfGFP	This study
pPB30.1 (391)	p415-GDP^PR^-Cox17-mCherry-sfGFP	This study
pPB21.1 (202)	p415-GDP^PR^-Pet191-mCherry-sfGFP	This study
pPB25.1 (386)	p415-GDP^PR^-Tim9-mCherry-sfGFP	This study
pPB28.1 (389)	p415-GDP^PR^-Tim10-mCherry-sfGFP	This study
pMaM98 (158)	p415-GDP^PR^-Ubi-I-mCherry-sfGFP	[51]
pMaM99 (159)	p415-GDP^PR^-Ubi-M-mCherry-sfGFP	[51]
pMaM100 (160)	p415-GDP^PR^-Ubi-F-mCherry-sfGFP	[51]

### Purification of ubiquitinated proteins via 6His-tagged ubiquitin

Yeast were transformed with plasmids that encoded 6His-tagged ubiquitin (6His-Ub) and Cox12_FLAG_ (or Cox12_FLAG_ variants as indicated). Corresponding empty vectors were used as controls. *GAL10* or *CUP1* promoters were used to express Cox12_FLAG_. The *CUP1* promoter was also used to express 6His-UB. In experiments in which Cox12_FLAG_ expression was driven by the *GAL10* promoter, yeast were grown in selective modified minimal medium without ammonium sulfate supplemented with 0.1% proline and 0.003% SDS, with glycerol as a carbon source at 28 °C or as indicated. For the final 6 h of growth, the yeast were collected by centrifugation (5 min at 3000×*g*) and resuspended in a small volume of media with galactose (0.5% *w*/*v*), CuSO_4_ (100 μM), and MG132 (75 μM) added. In the experiments in which Cox12_FLAG_ expression was driven by the *CUP1* promoter, yeast were grown in selective modified minimal medium without ammonium sulfate with glucose as a carbon source at 28 °C until the mid-log phase was reached. For the final 1 h, the yeast were collected by centrifugation (5 min at 3000×*g*) and resuspended in a small volume of media. CuSO_4_ (100 μM) and MG132 (75 μM) were added to the medium, and the cells were incubated at 28 or 37 °C. The cells were harvested by centrifugation for 3 min at 20,000×*g*. Cell pellets were then resuspended in denaturing lysis buffer (6 M guanidine hydrochloride, 100 mM KPi [pH 8.0], 10 mM Tris-HCl [pH 8.0], 50 mM iodoacetamide, 5 mM imidazole, 0.1% Triton X-100, 2 mM PSMF, and 75 μM MG132) and disrupted by 15 min of vortexing with glass beads. The solution was clarified by centrifugation for 10 min at 20,000×*g*, and the supernatant was moved to a new tube. Before the addition of nickel-nitrilotriacetic acid (Ni-NTA) agarose beads (Qiagen), load fractions were collected from the samples. The samples were incubated with Ni-NTA for 2 h at room temperature with gentle mixing. After centrifugation for 2 min at 200×*g*, the supernatant was removed from Ni-NTA beads. Unbound fractions were collected, and Ni-NTA beads were washed once with lysis buffer and three times with wash buffer (8 M urea, 100 mM KPi [pH 6.4], and 10 mM Tris-HCl [pH 6.4]). Purified proteins were eluted at 65 °C with 2× Laemmli buffer (4% SDS, 20% glycerol, 125 mM Tris-HCl [pH 6.8], and 0.02% bromophenol blue) supplemented with 100 mM DTT. Load and unbound samples were precipitated with 10% trichloroacetic acid (TCA), washed with acetone, air dried, and resuspended with 2× Laemmli buffer supplemented with 100 mM DTT. All of the samples were denatured at 65 °C.

### Import of radiolabeled proteins into isolated mitochondria

Mitochondria were isolated from wild-type cells that were grown in YPG medium at 24 °C. A standard protocol of the isolation by differential centrifugation was used [86]. Crude mitochondrial pellets were suspended in SM buffer (250 mM sucrose and 10 mM 3-morpholinopropane-1-sulfonic acid [MOPS]-KOH [pH 7.2]). Protein content was measured using the Bradford assay, and the concentration was adjusted to 10 mg of protein/ml. Isolated mitochondria were stored frozen at − 80 °C. For mRNA synthesis, the mMESSAGE mMACHINE SP6 Transcription Kit (Invitrogen) was used. For the synthesis of [^35^S]methionine-labeled proteins, the Flexi rabbit reticulocyte lysate translation system (Promega) or TNT quick coupled transcription/translation system (Promega) was used in accordance with the manufacturer’s protocols. At the end of the translation reactions, proteins were precipitated with ammonium sulfate and resuspended in urea buffer (8 M urea, 30 mM MOPS-KOH [pH 7.2], and 10 mM DTT). Radiolabeled proteins were incubated with isolated mitochondria in the import buffer (250 mM sucrose, 5 mM MgCl_2_, 80 mM KCl, 10 mM MOPS-KOH, 5 mM methionine, and 10 mM KH_2_PO_4_ [pH 7.2] supplemented with 2 mM ATP and 2 mM NADH) at 25 °C for the indicated time. Radiolabeled proteins comprised 2% of the import reaction mixture. Resulting dilution of urea allows for rapid self-folding of ubiquitin while Cox12 folding requires Mia40 assistance in the IMS [48–50, 87]. Import reactions were stopped by placing the samples on ice and adding IA to a final concentration of 50 mM. Where indicated, to remove non-imported proteins, the samples were treated with 10 μg/ml proteinase K on ice for 15 min. Protease digestion was stopped by the addition of phenylmethylsulfonyl fluoride (PMSF) to a final concentration of 2 mM. Mitochondria were then reisolated by centrifugation for 10 min at 20,000×*g* and washed with SM buffer supplemented with 2 mM PMSF. Finally, the samples were dissolved in 2× Laemmli buffer with the addition of 100 mM DTT or 50 mM IA for reducing and non-reducing SDS-polyacrylamide gel electrophoresis (PAGE), respectively. Radiolabeled proteins were detected by digital autoradiography using Storage Phosphor screens (GE Healthcare) and Typhoon TRIO+ Variable Mode Imager (GE Healthcare).

### Screens for factors involved in the degradation of tFT-tagged proteins

Query strains that expressed IMS proteins that were endogenously tagged with the mCherry-sfGFP timer were obtained from the tFT library [62]. Screens were performed essentially as described previously [62]. Briefly, the screens were conducted in 1536-colony format, with four technical replicates that were arranged next to each other on agar plates. Using synthetic genetic array methodology [88], each query strain was crossed to an array of haploid strains carrying knockout [89], temperature-sensitive (*ts*) [90], or DAmP [91] alleles of individual components of the ubiquitin-proteasome system. The selection of diploids, sporulation, and the selection of haploids that simultaneously carried a tFT-tagged protein and a genetic perturbation were performed by sequential pinning on appropriate selective media using pinning robots (BioMatrix, S&P Robotics) as described previously [92], followed by seamless marker excision [93]. Fluorescence intensities of the final colonies were measured in the mCherry channel (587/10 nm excitation, 610/10 nm emission) and sfGFP channel (488/10 nm excitation, 510/10 nm emission) after 24 h of growth on synthetic complete medium that lacked histidine at 30 °C using an Infinite M1000 or Infinite M1000 Pro plate reader equipped with stackers for automated plate loading (Tecan) and custom temperature control chambers.

Measurements were filtered for potentially failed crosses based on colony size after haploid selection. Fluorescence intensity measurements were log-transformed and corrected for spatial effects on plates by local regression. Median effects for tFT and deletion strains were subtracted from log-ratios of mCherry and sfGFP intensities. Standard deviations were regressed against the absolute fluorescence intensities. Changes in protein stability were divided by the regressed standard deviations, yielding a measurement comparable to a *z*-score, and tested against the hypothesis of zero change. *p* values were calculated using a moderated *t* test (R/Bioconductor package limma) and adjusted for multiple testing using the Benjamini-Hochberg method.

### Measurements of fluorescent signals in liquid culture

Wild-type yeast were transformed with plasmids that allowed the constitutive expression of tFT fusions and were grown in liquid selective media. The fluorescence intensities of mCherry and sfGFP proteins of the tFT were measured using an Infinite M1000 plate fluorimeter (Tecan) in 96-well plates directly from the culture. Cultures of yeast with an empty vector were used to correct for background fluorescence. Data were acquired using Magellan software (Tecan). All of the calculations were performed using Microsoft Excel software.

### Confocal microscopy

The acquisition of confocal images of live yeast cells was performed using a Zeiss LSM800 confocal microscope with a 63× 1.4 oil immersion objective and ZEN 2.3 software (Zeiss). Yeast that expressed selected IMS proteins that were tagged with tFT were grown in minimal synthetic media with glycerol as a main carbon source at 24 °C. Prior to imaging, growth media were supplemented with MitoTracker Deep Red FM (200 nM, Thermo Scientific) and Calcofluor White M2R (5 μg/ml, Sigma), followed by 10 min of incubation. For imaging, the samples were placed on poly-l-lysine-coated glass slides (Sigma). The acquisition settings were adjusted for each experimental condition for maximum intensity projections. Sixteen-bit images with a resolution of 358 × 358 pixels were acquired, exported to Adobe Photoshop software with adjustments of contrast and brightness, and assembled in Adobe Illustrator software.

### Homology modeling

The homology modeling of *S. cerevisiae* Tom40 was performed with the template of the *Neurospora crassa* Tom40 structure PDB:5O8O [67]. To align the sequences, MUSCLE alignment software was used [94]. The homology model was created with Modeller software [95]. The image was generated using Chimera software [96].

### Miscellaneous

Gene and protein names are used in accordance with the Saccharomyces Genome Database. Total protein cell extracts were made using the alkaline lysis protocol (adapted from Yaffe and Schatz) or by disrupting the cells with glass beads in the presence of precipitating agent (TCA) as described previously [97, 98]. Samples were denatured in 2× Laemmli buffer with 100 mM DTT at 65 °C. Samples were analyzed by SDS-PAGE followed by Western blot. The antibodies that were used for immunodetection were raised in rabbits and verified for specificity, with the exception of commercial mouse monoclonal anti-ubiquitin antibody (P4D1; Santa Cruz Biotechnology Cat# sc-8017, RRID:AB_628423; 1:500 dilution) and anti-FLAG M2 (Sigma-Aldrich Cat# F1804, RRID:AB_262044, 1:500 dilution) antibodies. The chemiluminescent signals were detected using X-ray films or an ImageQuant LAS4010 instrument (GE Healthcare). For Cox12_FLAG_ and its derivatives, we frequently detect additional band with altered slower migration speed. This additional band is specific to Cox12_FLAG_ protein as it can be detected both with anti-Cox12 and anti-FLAG antibodies. The additional band can be detected also in case of Cox12 variants without cysteine residues (Cox12_C-FREE-FLAG_) or without lysine residues (Cox12_K-FREE-FLAG_). For quantification of Western blot and autoradiography results, densitometry was performed using ImageQuant TL (GE Healthcare), and calculations were performed using Microsoft Excel software. The results of the quantifications are presented as mean from an indicated number of replicates (*n*). For *n* < 6 individual data points are provided in Additional file 2: Table S2. Error bars represent the standard error of the mean (SEM). Images were processed with Adobe Photoshop software and assembled into figures using Adobe Illustrator software. To identify highly evolutionarily conserved amino acid residues in *S. cerevisiae* Cox12 protein, the alignment of Cox12 sequences from 235 related fungi was performed using Clustal Omega [99]. Sequences that introduced gaps into the alignment were excluded. The data were visualized using WebLogo [100].

## Additional files

Additional file 1: **Table S1.** Results of screens for machinery involved in the degradation of IMS proteins. Changes in protein stability, represented as a *z*-score and corresponding *p* values, are provided for all combinations of the tested tFT-tagged IMS proteins and mutants of components of the ubiquitin-proteasome system. NA, not available. (XLSX 114 kb)

Additional file 2: **Table S2.** Individual data points for quantifications presented on Figs. 5a and 6c–e. Individual data points that were used for the preparation of the graphs. Sample names are as on corresponding figures. (XLSX 11 kb)
